# Skin testing with bendamustine: what concentration should be used?

**DOI:** 10.1186/s13223-020-00469-3

**Published:** 2020-07-30

**Authors:** Laura Malinauskiene, Kestutis Cerniauskas, Kotryna Linauskiene, Linas Griguola, Anzelika Chomiciene, Audra Blaziene

**Affiliations:** Faculty of Medicine, Institute of Clinical Medicine, Clinic of Chest Diseases, Immunology and Allergology, Vilnius University, Santariškių str. 2, LT-08661 Vilnius, Lithuania

**Keywords:** Bendamustine, Hypersensitivity reaction, Intradermal tests, Controls

## Abstract

We present a case of the patient, who developed hypersensitivity reaction during the treatment of chronic lymphocytic leukemia. Bendamustine was suspected as a culprit agent. The patient as well as 3 controls underwent skin testing with the concentrations of the bendamustine described in earlier studies. We doubted the testing recommendations as all the controls developed serious local reactions. The clinical meaning of the positive skin test reaction in the patient remained unclear and questioned the safeness of recommended testing concentrations as in certain situations wrong diagnosis could be made or even harm could be done. Future investigations are needed when allergy to bendamustine is suspected.

To the Editor:

Bendamustine is an alkylating agent used intravenously for the treatment of refractory hematologic or other malignancies (e.g., chronic lymphocytic leukemia (CLL) and B-cell non-Hodgkin lymphoma) with other agent or as monotherapy [1]. There a few reported cases where hypersensitivity reaction to this drug was reported and allergological work-up was carried out [2, 3]. We present a case of the patient, who developed hypersensitivity reaction during treatment of CLL and bendamustine was suspected. She as well as 3 controls underwent skin testing with the concentrations of the bendamustine described in earlier studies. All controls developed local reactions, so the clinical meaning of the positive skin test reaction in the patient remained unclear.

A 68-year-old woman, diagnosed with CLL received bendamustine tolerating the first two cycles. During the third cycle 3 h after bendamustine infusion she suffered from fever, chills, pruritus, hives, general malaise and hypotension (80/50 mmHg). Tryptase was obtained 12 h after reaction started, it was < 1.0 µg/l. Reaction disappeared within few hours after treatment with saline infusion, paracetamol, methylprednisone, noradrenaline and antihistamines.

After 3 weeks we performed skin-prick-test (SPT) at 1 mg/ml and intradermal testing (IDT) at 0.001, 0.01, 0.1 and 1 mg/ml, with immediate (at 20 min) and delayed (at 24 h and 5 days) readings. SPT was negative. IDT at 0.1 and 1 mg/ml were positive (infiltration, redness and papule) at 7–8 h, and remained positive for 6 weeks. As negative controls 3 physicians underwent SPT at 1 mg/ml with negative results and IDT with 0.1 and 1 mg/ml. These concentrations provoke cutaneous reactions in all controls (Fig. 1). Immediate reading showed slight erythema which was considered as toxic reaction but after 10 days infiltration and redness in the IDT site developed at 1 mg/ml, in one case with vesicles, also slight induration and redness developed at the sites of IDT with lower concentration. Skin biopsy was taken in order to differentiate the origin of the skin changes.

**Fig. 1:**
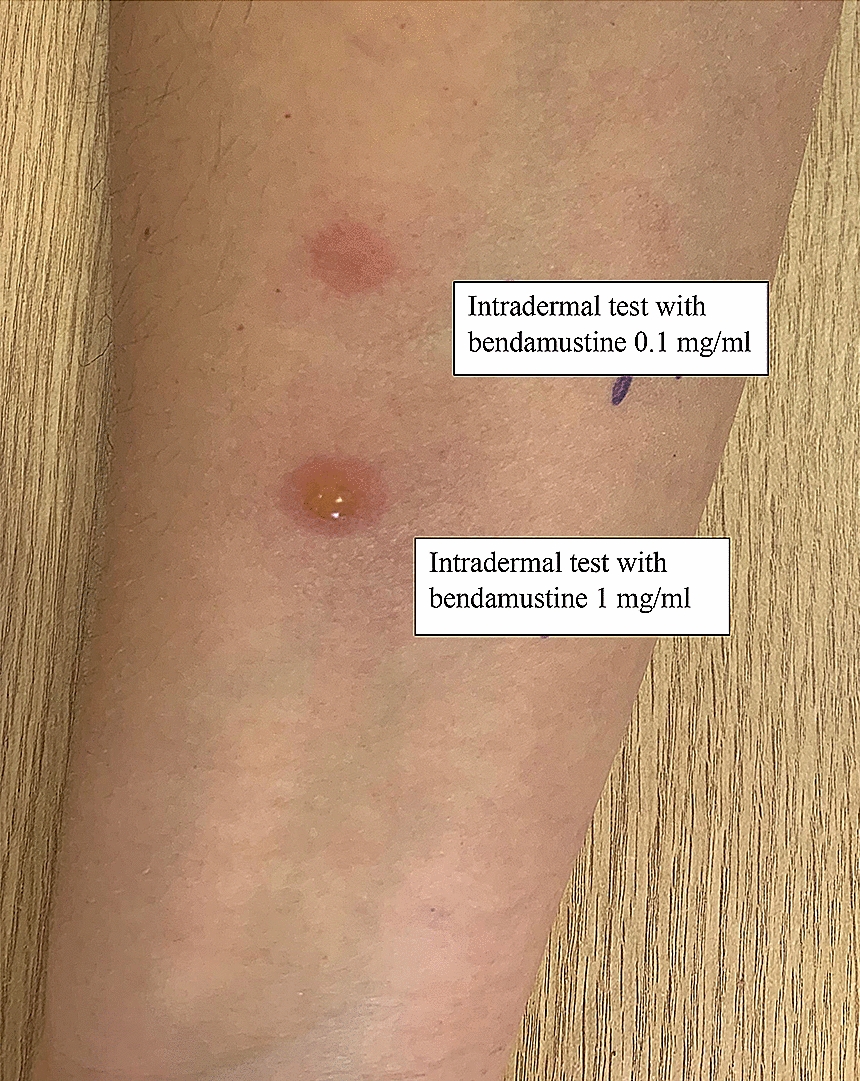
Cutaneous reaction to bendamustine at 0.1 and 1 mg/ml after 10 days in one of the control patient

Patohistology revealed slight hyperplasia of epidermis, moderate vacuolated and necrotic keratinocytes in the basal layer of epidermis, lymphocytic spongiosis. In the papillary dermis perivascular infiltration with PMN, edema on the endothelium, leucocytoclasis, signs of the exocytosis of lymphocytes. All changes were more pronounced in the specimen taken form 1 mg/ml site in comparison with 0.1 mg/ml. Conclusion of the pathological examination was as follows: junction dermatitis with the signs of vasculitis, most probable toxic skin damage.

Skin reactions in the controls persisted for 6 weeks, after this brown pigmentation remained visible for 4 months.

In this case we showed that it is risky to rely on positive skin reactions when non standardized skin concentrations are used. According to ENDA/EAACI Drug Allergy Interest Group position paper except for platinum salts, an IgE-mediated hypersensitivity to chemotherapeutic drugs has not been demonstrated [4]. The irritant potential of chemotherapeutic drugs appears to be low. For platinum salts, the use of undiluted drugs is recommended for skin testing. For other chemotherapeutic drugs, SPT with undiluted agents are probably nonirritant, but due to toxicity concerns, a general recommendation cannot be given. For IDT, a 1/10 dilution of most chemotherapeutic drugs is nonirritant and may be used in clinical practice (moderate/strong), whereas higher concentrations appear to be irritant. Some authors describe successful desensitization with bendamustine in case when hypersensitivity to bendamustine was diagnosed by skin testing without testing controls. Skin reactions including rash, toxic skin reactions and bullous exanthema to bendamustine were reported in clinical trials and during post marketing. More events occurred when bendamustine was used in combination with other anticancer drugs [5]. In our patient we obtained positive skin test reactions morphologically looking like a true allergic reaction, but our controls developed much more severe reaction. We carefully studied all steps of testing controls and virtually denied possibility of errors (e.g., incorrect dilution). In the earlier study 3 patients with CLL were used as controls and did not experienced any reaction on SPT and IDT with bendamustine [3]. We suggest that our patient was immunosuppressed because of her disease and treatments she received and this resulted in milder skin reaction. So it is unknown what control is more reliable—the one with the same disease or a healthy one. Nevertheless, patient was diagnosed as having allergy to bendamustine and her treatment was changed to ibrutinib. Testing controls is very helpful although it raises ethical issues as this procedure can be harmful and leave at least skin disfigurement. Skin biopsy in some cases can be helpful to differentiate between toxic and allergic reactions.

## Data Availability

Not applicable.

## Abbreviations

CLL: Chronic lymphocytic leukemia
SPT: Skin-prick-test
IDT: Intradermal testing
